# Spectrophotometric Methods for the Determination of Linagliptin in Binary Mixture with Metformin Hydrochloride and Simultaneous Determination of Linagliptin and Metformin Hydrochloride using High Performance Liquid Chromatography

**Published:** 2013-03

**Authors:** Ramzia I. El-Bagary, Ehab F. Elkady, Bassam M. Ayoub

**Affiliations:** 1 *Department of Pharmaceutical Chemistry, Faculty of Pharmacy, Cairo University, Kasr El-Aini St., Cairo 11562, Egypt*; 2 *Department of Pharmaceutical Chemistry, Faculty of Pharmacy, British University in Egypt, El-Sherouk city, Cairo 11837, Egypt*

**Keywords:** linagliptin, metformin hydrochloride, reversed-phase liquid chromatography, spectrophotometry, pharmaceutical preparation

## Abstract

Simple, accurate and precise Zero order, first derivative spectrophotometric and chromatographic methods have been developed and validated for the determination of linagliptin (LNG) and metformin HCl (MET). The zero order and first derivative spectrophotometric methods were used for the determination of LNG in the range of 5-30 μg mL^−1^ by measuring the absorbance at 299 nm and 311 respectively. Besides, a reversed-phase liquid chromatographic (RP-LC) method is described for the simultaneous determination of LNG and MET. Chromatographic separation was achieved on a Symmetry^®^ Waters C18 column (150 mm × 4.6 mm, 5 μm). Isocratic elution based on potassium dihydrogen phosphate buffer pH (4.6) - methanol (30:70, *v/v*) at a flow rate of 1 mLmin^−1^ with UV detection at 260 nm was performed. Linearity, accuracy and precision were found to be acceptable over the concentration ranges of 0.125-4 μg mL^−1^ and 20-800 μg mL^−1^ for LNG and MET, respectively. The results were statistically compared using one-way analysis of variance (ANOVA). The optimized methods were validated and proved to be specific, robust, precise and accurate for the quality control of the drugs in their pharmaceutical preparation.

## INTRODUCTION

**Figure 1:**
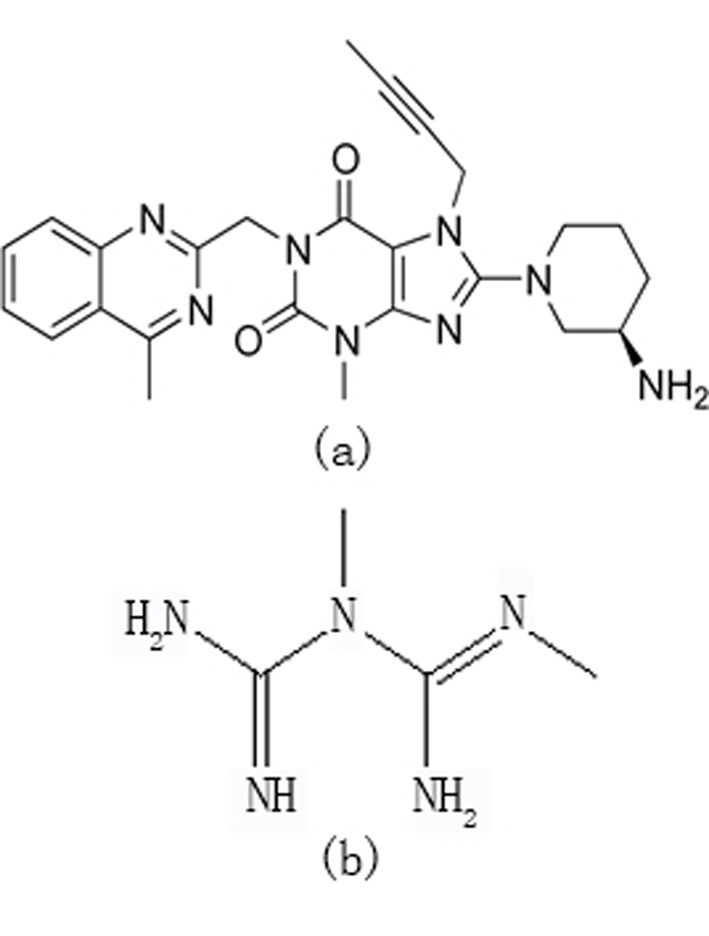
Chemical structures of Linagliptin (a) and metformin (b)

Linagliptin (LNG), 8-[(3*R*)-3-aminopiperidin-1-yl]-7-(but-2-yn-1-yl)-3- methyl-1-[(4-methylquinazolin-2-yl)methyl]-3,7-dihydro-1*H*-purine-2,6-dione] (Fig. 1) is a novel hypoglycemic drug that belongs to dipeptidyl-peptidase-4 inhibitor class (1, 2). DPP-4 inhibitors represent a new therapeutic approach to the treatment of type 2 diabetes that functions to stimulate glucose-dependent insulin release and reduce glucagon levels. This is done through inhibition of the inactivation of incretins, particularly glucagon-like peptide-1 (GLP-1) and gastric inhibitory polypeptide (GIP), thereby improving glycemic control (3). Recently, DPP-4 inhibitors have been recommended in the treatment of diabetes mellitus to improve glycemic control (4) and it is effective in controlling the metabolic syndrome and resulted in significant weight loss, a reversal of insulin resistance, islet and adipocyte hypertrophy, and alleviated hepatic steatosis (5). Only two methods have been described for the determination of LNG in its pharmaceutical preparation based on reversed-phase liquid chromatography (6, 7).

Metformin (MET), N, N- dimethylimidodicarbonimidic diamide (Fig. 1b) is a biguanide hypoglycemic drug that stimulates glycolysis in peripheral tissues and it is regarded as the main component in mixed therapies of oral hypoglycemic. Literature survey reveals some methods for the determination of MET in mixtures including LC/MS/MS (8) and HPLC (9-12).

There is no method reported in the literature for the simultaneous determination of LNG and MET in their binary mixture. Thus, the aim of the present work was to develop different spectrophotometric method for the determination of LNG in its binary mixture with MET. Spectrophotometry continues to be very popular, because of its simplicity and low cost so it has long been applied for the analysis of many drugs (13-19). Besides, it would have been valuable to develop and validate simple RP-LC method that could be applied for the simultaneous determination of LNG and MET in their binary mixture.

## EXPERIMENTAL

### Instrumentation

A Jenway 6800 double beam ultraviolet/visible spectrometer, UK, connected to an IBM compatible computer with 1-cm quartz cell and supported with Jenway flight deck software was used. The HPLC system consisted of a Schimadzu LC-20 AT Liquid Chromatograph (Japan) using a Symmetry^®^ Waters C18 column (150 mm × 4.6 mm, 5 μm) (Ireland). The system was equipped with a UV-visible detector (SPD-20A, Japan) and an autosampler (SIL-20A, Schimadzu, Japan). An Elma S100 ultrasonic processor model KBK 4200 (Germany) was used for the degassing of the mobile phases.

### Reagents and reference samples

Pharmaceutical grade LNG, certified to contain 99.80%, pharmaceutical grade MET, certified to contain 99.80% and Jentadueto^®^ tablets nominally containing 2.5 mg of LNG and 500 mg of MET per tablet were supplied from Boehringer Ingelheim company, USA. HPLC grade methanol was purchased from Fisher Scientific (Loughborough, Leicestershire, UK). Potassium dihydrogen phosphate was purchased from VWR Chemicals (Pool, England). Bi-distilled water was produced in-house (Aquatron Water Still, A4000D, UK). Membrane filters 0.45 µm from Whatman international Ltd., (Maidstone, UK) were used. All other chemicals and reagents used were of analytical grade unless indicated otherwise. Standard stock solutions of each drug (1 mgmL^-1^) were prepared by dissolving 100 mg of the drug in methanol in a 100 mL volumetric flask and then completed to volume with methanol. Then required concentrations were prepared by serial dilutions with methanol of these stock solutions.

### Chromatographic conditions

Chromatographic separation was achieved on a Symmetry^®^ Waters C18 column (150 mm × 4.6 mm, 5 μm). Isocratic elution using a mobile phase consisting of potassium dihydrogen phosphate buffer pH (4.6) - methanol (30:70, *v/v*) with UV detection at 260 nm was performed. The buffer solution was filtered through 0.45 µm membrane filter and degassed for 30 min in an ultrasonic bath prior to use. The mobile phase was pumped through the column at a flow rate of 1 mL min^-1^. Analyses were performed at ambient temperature and the injection volume was 25 µL.

### Samples’ preparation

Twenty tablets were weighed and the coats were removed by carefully rubbing with a clean tissue wetted with methanol. Fifty milliliters of methanol were added to an accuratelyweighed amount of the finely powdered Jentadueto^®^ tabletsequivalent to 500 mg of MET and 2.5 mg of LNG, sonicated for 15 min and then made up to 100 ml with methanol. The solutions were filtered followed by serial dilution to the required concentrations for each experiment.

### Procedures

**Zero order spectrophotometric method.** Aliquots from LNG stock standard solution equivalent to 50–300 μg were accurately measured and transferred into sets of 10 mL volumetric flasks and then completed to volume with methanol. The zero order absorption of each solution was recorded against methanol as a blank at 299 nm, then plottedagainst its corresponding concentration and the regressionparameters were computed.

**First derivative spectrophotometric method.** Aliquots from LNG stock standard solution equivalent to 50–300 μg were accurately measured and transferred into sets of 10 mL volumetric flasks and completed to volume with methanol. The zero order absorption spectraof each solution were recorded against methanol as a blank in the range 200-350 nm, and then the first derivative spectra were computed. The amplitude at 311 nm was measured for LNG then plotted against corresponding concentrations and the regressionparameters were computed.

**Liquid chromatography linearity and repeatability.** Accurately measured aliquots of working standard solutions equivalent to 1.25-40 µg LNG and 200-8000 µg MET were separately transferred into two series of 10 mL volumetric flasks and then completed to volume with methanol. A volume of 25 µL of each solution was injected into the chromatograph. The chromatographic conditions mentioned under [Chromatographic conditions], including the mobile phase at a flow rate 1 mL min^-1^, detection at 260 nm and run time program for 10 min were adjusted. A calibration curve for each compound was obtained by plotting area under the peak (AUP) against concentration (C). The repeatability of the method was assessed by analyzing a mixture containing 2.5 µg, 500 µg of LNG and MET, respectively (*n*=6). The precision (%R.S.D) for each compound was calculated.

**Assay of laboratory-prepared mixtures.**

***Zero order and first derivative methods*.** The absorption spectrum was recorded for the laboratory prepared mixtures containing MET and LNG, against methanol as a blank. The zero order absorption spectraat 299 nm were used for the direct determination of LNG. The amplitudes of the first derivative spectra of the laboratory prepared mixtures containing MET and LNG were measured at 311 nm for LNG. The concentrations of LNG were calculated from their corresponding regression equations.

***HPLC method.*** The procedure mentioned under [Liquid chromatography linearity and repeatability] was repeated using laboratory prepared mixtures equivalent to 0.5-3.5 µg mL^-1^ LNG and 100-700 µg mL^-1^ MET. The concentration ratio of LNG to MET in the mixtures was 0.5%, *w/w* as in the tablet dosage form. The concentrations of the examined drugs were calculated by the calibration equations.

***Assay of Jentadueto^®^ tablets.*** Twenty tablets were weighed and the coats were removed by carefully rubbing with a clean tissue wetted with methanol. Fifty milliliters of methanol were added to an accuratelyweighed amount of the finely powdered Jentadueto^®^ tabletsequivalent to 500 mg of MET and 2.5 mg of LNG, sonicated for 15 min and then made up to100 ml with methanol. The solutions were filtered followed by serial dilution to the required concentrations for each experiment. The procedure was continued as mentioned under general procedures and calibration.

## RESULTS AND DISCUSSION

Literature survey reveals that only two liquid chromatographic methods have been developed for the determination of LNG (6, 7) and that there has not been any published work for the determination of LNG and MET in binary mixtures. Thus, the development of spectrophotometric and chromatographic methods for the determination of LNG in its binary mixture with MET was of interest.

### Methods’ development

**Figure 2:**
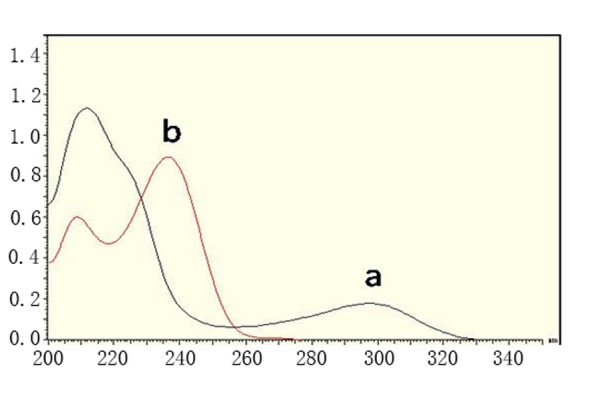
Zero order spectra of linagliptin 5 μg.ml^-1^ (a) and metformin hydrochloride 12 μg.ml^-1^ (b)

**Figure 3:**
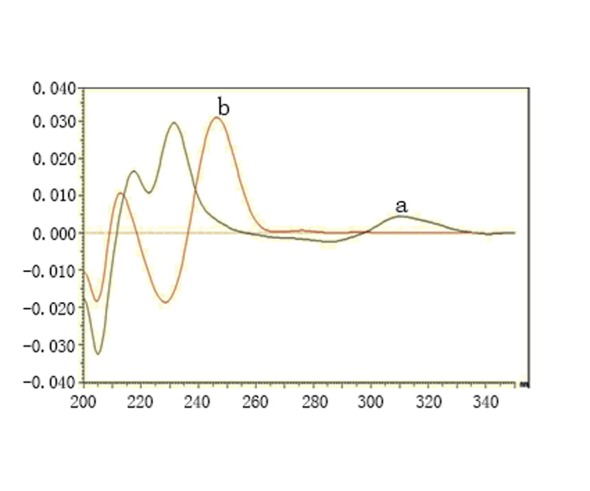
First derivative spectra of linagliptin 5 μg.ml^-1^ (a) and metformin hydrochloride 12 μg.ml^-1^ (b)

**Spectrophotometric methods.** LNG could have been determined using zero order and first derivative spectrophotometry without interference from MET (Fig. 2 and Fig. 3) and good results were obtained (Table 1 and Table 2). First derivative spectra were obtained from the same spectra of zero order with good linearity and accuracy. Direct UV-absorbance measurement and derivative spectrophotometry are well established techniques for the assay of drugs in mixtures and in pharmaceutical dosage forms enhancing the resolution of overlapping bands. It can be applied for the determination of a drug in the presence of another by selecting a wavelength where contribution of one compound is almost zero while the compound to be determined has a reasonable value, so it has been used in the determination of many drugs (13-19).

**Table 1:** Results obtained by zero order method for the determination of linagliptin in its binary mixture with metformin

λ_max_ of measurements	299 nm
Obedience of Beer’s law	5-30 μg.ml^-1^
Regression equation	A_299_=0.0348 C_μg/ml_ - 0.0021
Regression coefficient (r^2^)	0.9999
LOD μg.ml^-1^	0.23
LOQ μg.ml^-1^	0.78
S_b_	1.3 × 10^-4^
S_a_	0.003
Confidence limit of the slope	0.0348 ± 1.04 × 10^-4^
Confidence limit of the intercept	-0.0021 ± 2.7 × 10^-7^
Standard error of the estimation	0.0027
**Results**	
Drug in laboratory prepared mixture with MET	99.53 ± 1.07
Drug in combination with MET dosage form (JENTADUETO^®^)	99.33 ± 1.31
Drug added to the mixture	99.70 ± 1.16

**Table 2:** Results obtained by the first derivative method for the determination of linagliptin in its binary mixture with metformin

λ_max_ of measurements	311 nm
Obedience of Beer’s law	5-30 μg.ml^-1^
Regression equation	H_311_=0.0009 C_μg/ml_ - 0.0001
Regression coefficient (r^2^)	0.9995
LOD μg.ml^-1^	0.77
LOQ μg.ml^-1^	2.34
S_b_	3 × 10^-5^
S_a_	7.2 × 10_-4_
Confidence limit of the slope	0.0009 ± 6.45 × 10^-7^
Confidence limit of the intercept	-0.0001 ± 3 × 10^-9^
Standard error of the estimation	6.3 × 10^-4^
**Results**	
Drug in laboratory prepared mixture with MET	100.36 ± 1.17
Drug in combination with MET dosage form (JENTADUETO ^®^)	100.36 ± 1.91
Drug added to the mixture	100.00 ± 1.43

**HPLC method.** MET could not be determined by the previously mentioned methods as its absorption spectrum exhibits unresolved severe overlap from that of LNG (Fig. 2 and Fig. 3). So a chromatographic method has been applied to allow the simultaneous determination of the two drugs. HPLC greatly reduces the analysis time and allows for the determination of many individual components in a mixture using one single procedure (19).

**Figure 4:**
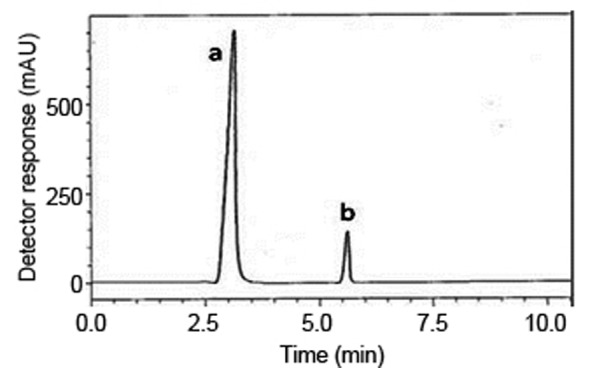
A typical LC chromatogram of 25 μL injector of Jentadueto^®^ sample solution, (a) metformin hydrochloride (500 μg.ml^-1^) and (b) linagliptin (2.5 μg.ml^-1^)

Various reversed-phase columns, isocratic mobile phase systems were attempted. Isocratic elution based on potassium dihydrogen phosphate buffer pH (4.6)-methanol (30:70, %*v/v*) was found optimum for the resolution and peak shapes. Minimum retention times were obtained at a flow rate 1 mL min^-1^. The UV detector was operated at 260 nm where good detector sensitivity was achieved. The retention times were 3.1 and 5.7 min for MET and LNG, respectively; as presented in Fig 4.

### HPLC system suitability tests

According to USP (20), system suitability tests are an integral part of liquid chromatographic methods in the course of optimizing the conditions of the proposed method. In the proposed LC method, system suitability tests were used to verify that resolution and reproducibility were adequate for analysis performed. Different parameters affecting the chromatographic separation were studied. The parameters of this test are column efficiency (number of theoretical plates), tailing of chromatographic peak, peak resolution factor, and repeatability as %R.S.D of peak areas for six injections and reproducibility of retention times. The results of these tests are listed in Table 3.

**Table 3:** System suitability tests for LC-UV method for the simultaneous determination of linagliptin and metformin hydrochloride in their binary mixture

Item	LNG	MET

N	3136	511
R	5.7	
T	1.01	1.06
RSD% of 6 injections		
Peak area	0.22	0.24
Retention time	0.31	0.38

N, number of theoretical plates); T, tailing of chromatographic peak); R, peak resolution factor); and repeatability as %R.S.D of peak area for six injections and reproducibility of retention as %R.S.D of retention time.

### HPLC Method validation

**Linearity.** Linearity was studied for LNG and MET. A linear relationship between area under the peak (AUP) and component concentration (C) was obtained. The regression equations were also computed. The linearity of the calibration curves were validated by the high value of correlation coefficients. The analytical data of the calibration curves including standard deviations for the slope and intercept (S_b_, S_a_) are summarized in Table 4.

**Table 4:** Results obtained by LC-UV method for the simultaneous determination of linagliptin and metformin hydrochloride in their binary mixture

Item	Linagliptin	Metformin hydrochloride

Retention time	5.7	3.1
Wavelength of detection	260 nm	260 nm
Range of linearity	0.125-4 μg mL^-1^	25-800 μg mL^-1^
Regression equation	Area × 10^-4^ = 4.7021 C _μg mL^-1^_ + 0.0312	Area × 10^-5^ = 0.0591 C _μg mL^-1^_ + 0.0859
Regression coefficient (r^2^)	0.9997	0.9998
LOD (μg mL^-1^)	0.03	5.72
LOQ (μg mL^-1^)	0.09	19.08
S_b_	0.013	1.7 × 10^-4^
S_a_	0.03	0.08
Confidence limit of the slope	4.7021 ± 0.14	0.0591 ± 4.7 × 10^-3^
Confidence limit of the intercept	0.0312 ± 4.1 × 10^-4^	0.0859 ± 1.5 × 10^-5^
Standard error of the estimation	0.043	0.114
**Precision**		
Intraday %R.S.D	0.16-0.36	0.09-0.42
Interday %R.S.D	0.17-1.13	0.10-0.89
Drug in dosage form	100.07 ± 1.42	99.64 ± 1.41
**Accuracy**		
Drug in laboratory mixture	99.90 ± 1.74	100.42 ± 1.23
Drug added	100.56 ± 1.56	100.12 ± 1.36

**Accuracy.** Accuracy of the results was calculated by % recovery of 5 different samples of the laboratory prepared mixtures of LNG and MET and also by standard addition technique for Jentadueto^®^ tablet. The results obtained including the mean of the recovery and standard deviation are displayed in Table 4.

**Precision.** The repeatability of the method was assessed by analyzing 2.5 µg mL^-1^ of LNG and 500 µg mL^-1^ of MET (*n*=6). The values of the precision (%R.S.D) of repeatability and inter-day and intra-day precision (using 3 different concentrations in triplicates for three days) are displayed in Table 3 and Table 4.

**Specificity.** Specificity is the ability of the analytical method to measure the analyte response in the presence of interferences including degradation products and related substances. In the present work, specificity was checked by analyzing LNG with MET in laboratory prepared mixtures. Good resolution and absence of interference between drugs being analyzed are shown in Fig. 4. Besides, the chromatograms of the pharmaceutical formulation samples were checked for the appearance of any extra peaks. No chromatographic interference from any of the excipients was found at the retention times of the examined drugs (Fig. 4). In addition, the chromatograms of the drugs in the samples’ solutions were found identical to the chromatograms received by the standard solutions at the wavelengths applied. Moreover, good results were obtained for the determination of the cited drugs in the dosage form, Table 4. These results confirm the absence of interference from other materials in the pharmaceutical formulations and therefore confirm the specificity of the proposed method.

**Robustness.** The most important parameter to be studied was the resolution factor between the two peaks of LNG and MET. The flow rate of the mobile phase was changed from 1 mLmin^-1^ to 0.8 mLmin^-1^ and 1.2 mLmin^-1^, where resolution factors obtained were 5.70, 5.62 and 5.68 respectively. The organic strength was changed by % ± 2 where resolution factors obtained were 5.70, 4.86 and 5.81 respectively. Finally, a value of pH of the phosphate buffer was varied from 4.6 to 4.5 and 4.7, where resolution factors obtained were 5.70, 5.61 and 5.84 respectively. As can also be seen from these results, good values of the resolution factor were obtained for all these variations, indicating good robustness of the proposed LC method.

**Limit of detection and limit of quantification.** Limit of detection (LOD) which represents the concentration of analyte at S/N ratio of 3 and limit of quantification (LOQ) at which S/N is 10 were determined experimentally for the proposed methods and results are given in Table 4.

### Quantification, accuracy and precision for the zero order and the first derivative methods

Standard calibration curves were prepared by separately preparing series of different concentrations of LNG and applying the suggested procedures. The linearity of the calibration curves were validated by the high value of correlation coefficients. The analytical data of the calibration curves including standard deviations for the slope and intercept (S_b_, S_a_) are summarized in Table 1 and Table 2. The regression equations of these calibration graphs were utilized for the determination of concentrations of LNG in laboratory prepared mixtures and tablets. The reproducibility and accuracy of the suggested methods were assessed using different laboratory prepared solutions of different concentrations and determination of the concentrations in tablets. The results obtained were of good accuracy and precision. The applicability of the procedures for estimation of tablets was validated using standard addition technique as a check for accuracy (Table 1 and Table 2).

### Statistical analysis

Statistical analysis of the results obtained by the proposed methods and the reference methods for each drug were carried out by “SPSS statistical package version 11”. The significant difference between the reference methods and the described methods was tested by one way ANOVA (F-test) at *p*=0.05 as shown in Table 5 and Table 6. The test ascertained that there was no significant difference among the methods.

**Table 5:** Statistical comparison between the results of the proposed methods and the refernce method for the determination of linagliptin in binary mixture with metformin hydrochloride

Statistical term	Reference Method^b^	Zero order Method	First derivative Method	HPLC Method

Mean	99.45	99.53	100.36	99.90
S.D. ±	1.34	1.07	1.17	1.74
S.E. ±	0.60	0.48	0.52	0.78
%RSD	1.35	1.08	1.17	1.74
n	5	5	5	5
V	1.80	1.14	1.37	3.03
t (a2.306)				

a Figures in parentheses are the theoretical t value at (*p*=0.05). No significant difference between groups by using one way ANOVA with F equals 0.471 and p equals 0.707;

b Reference method: aliquots of standard solutions in methanol containing 1-6 μg/ml LNG were measured at 226 nm using methanol as a blank (2).

**Table 6:** Statistical comparison between the results of the proposed method and the refernce method for the determination of metformin hydrochloride in binary mixture with linagliptin

Statistical term	Reference Method^b^	HPLC Method

Mean	100.4	100.42
S.D. ±	0.28	1.23
S.E. ±	0.13	0.55
%RSD	0.28	1.22
n	5	5
V	0.08	1.51
t (a2.306)		0.04

a Figures in parentheses are the theoretical t value at (*p*=0.05). No significant difference between groups by using one way ANOVA with F equals 0.001 and p equals 0.973;

b Reference method for the spectrophotometric determination of metformin in the indian pharmacopeia (22).

## CONCLUSION

The proposed methods have the advantages of simplicity, precision, accuracy and convenience for the quantization of LNG in the presence of MET and also simultaneous determination of LNG and MET in their pharmaceutical dosage form. The three methods can be applied for the determination of the cited drugs in pharmaceutical dosage forms. The three methods were validated showing satisfactory data for all the method validation parameters tested. The developed methods can be conveniently used by quality control laboratories.
